# Management for A Depressive Patient with Femoral Neck Fracture by Electroconvulsive Therapy during COVID-19 Pandemic: A Case Report and Literature Review

**DOI:** 10.3390/ijerph20054004

**Published:** 2023-02-23

**Authors:** Nien-Mu Chiu, Ellen Yu-Lun Tseng

**Affiliations:** Department of Psychiatry, Kaohsiung Chang Gung Memorial Hospital, Chang Gung University College of Medicine, Kaohsiung 83301, Taiwan

**Keywords:** depression, electroconvulsive therapy, femoral neck fracture, shared decision-making, COVID-19 pandemic

## Abstract

Electroconvulsive therapy (ECT) is an effective treatment for refractory major depressive disorder with suicidal ideation. The most common adverse medical events are transient retrograde amnesia, falls and pneumonia. Hip fractures, associated with high-energy trauma by convulsions, were occasionally reported in western countries, in the period before the COVID-19 pandemic. Strict COVID-19 regulations influenced the course and further investigation of the treatment of post-ECT complications. A 33-year-old man, previously diagnosed with major depressive disorder, had a history of nine successful sessions of ECT treatment for depression five years ago. He was hospitalized again for 12 sessions of ECT for recurrent depression. Unfortunately, an ECT-induced right hip–neck fracture was noted after the ninth session of ECT, in March 2021. After receiving close reduction and internal fixation of the right femoral neck fracture, with three screws, his original daily function was restored. His treatment was regularly followed up at the outpatient clinic for 20 months; he achieved partial remission with three combined antidepressants. This case of ECT-induced right hip–neck fracture informed psychiatric staff to be aware of this rare adverse complication and ensure its appropriate management, especially during the COVID-19 pandemic.

## 1. Introduction

Electroconvulsive therapy (ECT) is a well-established, effective standard treatment of major depressive disorder with suicidal ideation [1]. However, ECT is one of the most controversial treatments in psychiatry [2]. There are many reports about the adverse effects of ECT, which vary in incidence according to different types of data, such as time periods, countries, hospitals, patient groups, treatment strategies, and defined surveillance policies. Some adverse effects are usually mild and transient and so have been less systematically studied than cognitive adverse effects. Common adverse events [3] include: transient memory impairment (75%, almost all patients are back to their cognitive baselines after six months) [4]; intra-ECT blood pressure surge; headache (from 26% to 85%) [5]; myalgia or back pain (31.4%, may be due to depolarizing muscle relaxants/convulsion/both); marked confusion (up to 10% of patients within 30 min of the seizure); delirium or agitation (from 5% to about 9%, shortly after the seizure, while the patient was coming out of anesthesia); long-term patchy retrograde amnesia (9.3% of patients complained of persistent memory difficulties); nausea; vomiting; self-limiting dry mouth (may be due to anticholinergic drugs in the premedication); teethache; anesthetic and cardiovascular complications; fractures of nonspinal bones or musculoskeletal injury (3.5% of unmodified ECT) [6]; fracture of vertebrae (from 20% to about 40% [7,8] versus 2% [9,10] of unmodified ECT in different reports); oral mucosa eruption; lockjaw, prolonged seizure (seizures lasting more than 180 s); falls (2.7 and 5.5 per 10,000 ECT treatments); pneumonia (1.8 and 3.8 per 10,000 ECT treatments, in a large epidemiological study in Canada) [11]; and death (0.002% per treatment and 0.01% for each patient) [4,11,12]. Hip fracture has rarely been reported as associated with high-energy trauma or convulsions [12,13].

The COVID-19 pandemic hit our ECT services hard. Under hospital regulations, only patients with a negative reverse transcriptase-polymerase chain reaction (RT-PCR) test, before hospitalization, were admitted to our psychiatric ward during the COVID-19 pandemic. Moreover, inpatients were not allowed to leave the ward during their hospital stay. All visitors were prohibited during the patient’s hospitalization. The number of patients undergoing ECT was limited to less than six patients each day. The strict limitations during the severe period of the COVID-19 pandemic impeded further investigations for post-ECT muscle cramps, which delayed the diagnosis of a femoral neck fracture.

Anesthetic agents and muscle relaxants are commonly used in the United States and Europe to reduce the negative effects of ECT and reduce the risk of injury, such as fractures. However, in numerous countries, such as Japan, Spain, and Russia, the practice of using modified ECT has been performed without muscle relaxants [14]. At our hospital, the use of ECT with muscle relaxants is not the standard protocol, but they are used, when necessary, as in this case. The use of muscle relaxants during ECT may delay the awakening times and increase the risk of choking or falling, necessitating more observation time and manpower when nursing patients. To our knowledge, there has been no previous report of hip–neck fracture, as a side effect of ECT, in Taiwan. In this paper, the authors describe an adult patient with major depressive disorder with suicidal ideation, who had been treated with bilateral temporal ECT, 12 times and later developed a right hip–neck fracture after his ninth ECT session. The authors report the only case of a femoral neck compression fracture that occurred in all cases that received ECT without muscle relaxants at our hospital in the past 30 years, from 1993 to 2023. 

## 2. Case Presentation

A 28-year-old male policeman had an acute episode of a major depressive disorder with suicidal ideation, influencing him to want to jump from a high building, despite receiving regular antidepressant treatment at two local clinics, for six months. He had received nine sessions of bilateral temporal ECT, with a frequency of three sessions per week. He had shown a good response to severe depressive episodes with a combined treatment of Wellbutrin Sustained-Release (SR) 300 mg daily, Propranolol 20 mg daily, and Clonazepam 1 mg daily, during his first hospitalization in 2016. He also had a comorbid history of hypothyroidism and irritable bowel syndrome, treated with the daily use of Thyroxine 0.05 mg and Otilonium bromide 120 mg. During the five years after discharge, he was treated with various antidepressants for fluctuating depression, including Escitalopram 20 mg daily, Duloxetine 120 mg daily, Agomelatine 50 mg daily, Mirtazapine 30 mg daily, and combined episodic augmentation with antipsychotic drugs, such as Quetiapine 25 mg daily or Aripiprazole 10 mg daily with a duration of a few months, which was partially effective. However, he had recurrent symptoms of major depression and received 20 sessions of self-paid repetitive transcranial magnetic stimulation (r-TMS), with a limited response, 14 months before the second hospitalization.

In 2021, he requested hospitalization for another 12 sessions of ECT, with an initially scheduled frequency of three times per week. However, the psychiatric ward had strict limitations regarding contact with families during severe the COVID-19 pandemic in Taiwan. Thus, after six sessions of ECT, the patient requested an early completion of the ECT course and early discharge from the hospital. After a discussion with the doctor, the frequency of ECT was shifted from Quaque Qmni Die, every other day (QOD) to every day (QD) on weekdays. Unfortunately, three days later after the ninth ECT session, a severe painful cramp in the right leg and difficulty walking were noted. The patient recalled that, after receiving ECT, at least one nurse accompanied him until he was alert, and he did not have any complaints about post-ECT amnesia. The nurses and patient all denied any incidence of his falling. ECT was performed using a Thymatron system IV machine with the following settings for this patient: pulse width, 0.5 ms; frequency, 60 Hz; duration, 0.6/0.9 s; current, 900 milliamps; energy, 34.7/50.4 mC; seizure time, 5/41 s under Thiamylal sodium 120 mg IV. His symptoms were initially mistakenly diagnosed as a painful muscle sprain and treated with conservative oral analgesics, an intramuscular analgesics injection and suspension of ECT for five days. Later, the remaining three sessions of ECT were performed QOD, under a muscle relaxant succinylcholine injection, immediately before ECT treatment. He was discharged with an additional diagnosis of ECT-induced myofascial pain, without X-ray examination.

**Figure 1:**
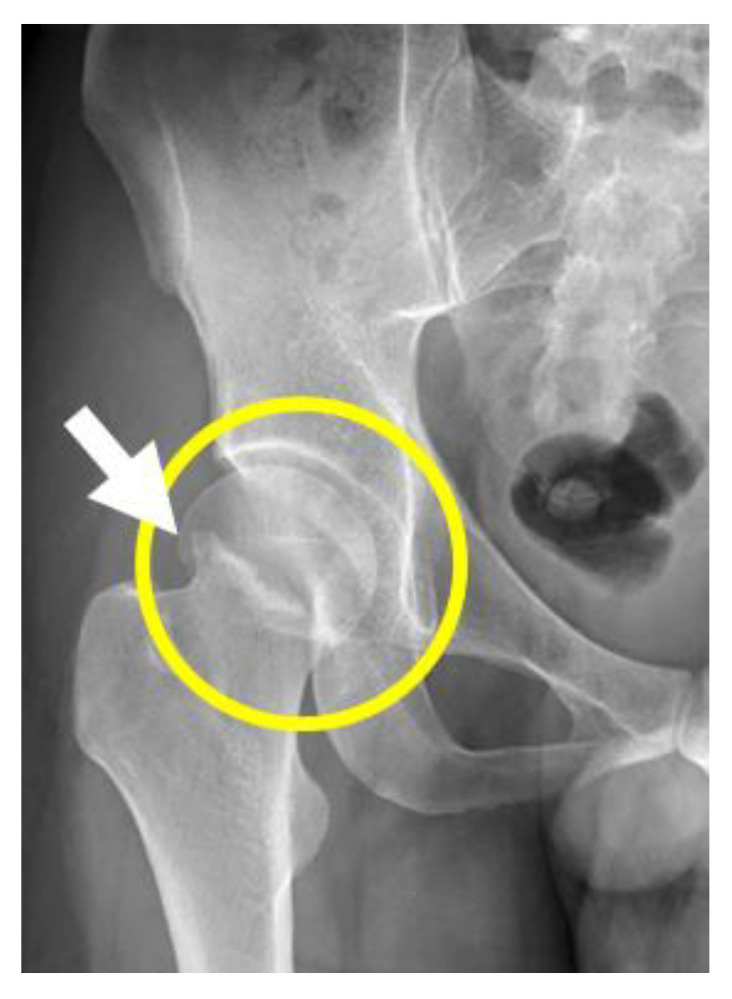
Lateral view X-ray of the right hip femoral neck fracture, Garden type I.

**Figure 2:**
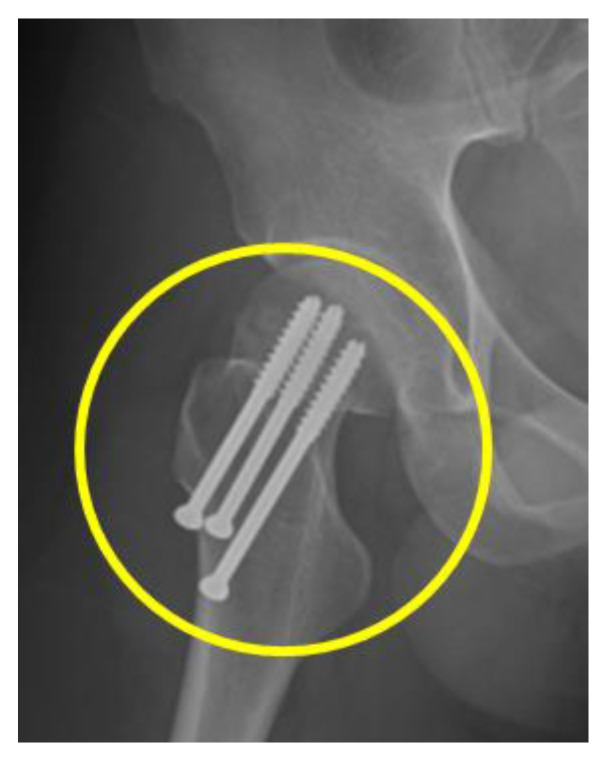
Lateral view X-ray of right hip femoral neck fracture, Garden type I post internal fixation of right femoral neck fracture with 6.5 mm cannulated screws × 3.

After the patient’s discharge from the hospital, the femoral neck fracture was confirmed by X-ray at another local hospital. However, different surgical recommendations were suggested at several hospitals. He returned to the psychiatric outpatient clinic again for further management. A Pelvic X-ray confirmed a right hip femoral neck fracture, of Garden type I, as shown in Figure 1, and he was then referred to an orthopedic doctor. Five weeks later, he was successfully treated by closed reduction and internal fixation of the right femoral neck fracture with three, 6.5 mm cannulated screws, as shown in Figure 2. One month after the surgery, he could walk again and returned to his original job. He was regularly followed up at the outpatient clinic and mainly treated with three combined antidepressants, namely, Wellbutrin SR 300 mg daily, Escitalopram 20 mg daily, and Agomelatine 50 mg daily for approximately 24 months.

A severity score of 27 points was recorded from of a Patient Health Questionnaire-9 (PHQ-9) [5] during the patient’s second hospitalization. After 20 months, the PHQ-9 score improved by 44% (from 27 points to 16 points). At an outpatient clinic follow-up, the Visual Analog Scale score for pain measurement decreased to 0/10 after a half month in remission.

## 3. Discussion

To our knowledge, this might be the first report from Taiwan showing that a hip fracture can occur during ECT treatment. Post-ECT hip fracture is rare, but it has been previously reported in other countries, the number of occurrences decreasing with the application of a muscle relaxant [15]. This patient did not receive muscle relaxant injections in the first nine sessions of ECT and when the frequency was increased from three times per week to every weekday. The possible mechanisms for causing injury include repeated strong muscle contractions and high-energy velocity trauma. The patient’s injury was initially mistaken as myofascial pain and was partially relieved by analgesic injections. In retrospect, the psychiatrists should have considered the possibility of a hip–neck fracture and urgently arrange a pelvic X-ray for early diagnosis, if indicated. If a pelvic X-ray could not have been arranged, due to the strict restrictions during the COVID-19 pandemic, a portable X-ray should have been considered.

The patient could recall fractures being mentioned as a possible complication when signing the informed consent form. Shared decision-making before the ECT treatment alleviated the psychological impact, and possible legal problems that could have arisen in this case [16]. Moreover, good therapeutic rapport, an apology and appropriate management of the ECT-induced complications were each of paramount importance during the course of the treatment. The COVID-19 pandemic had a negative impact on the provision of ECT services in Taiwan, as in other countries, such as Australia and Singapore [17] and India [18]. Contact with any family member was forbidden during the patient’s stay at the psychiatric hospital ward. The patient requested an early discharge, due to the strict restrictions in the ward during the COVID pandemic, and the frequency of his ECT treatment was changed from every other day to every weekday. However, the hip–neck fracture pain persisted despite him receiving muscle relaxant injections and switching back to the original frequency of treatment, for consequent ECT sessions. Fortunately, the complication could be corrected by appropriate orthopedic intervention using closed reduction and internal fixation of the right femoral neck fracture, with three cannulated screws. The outcome was successful for the patient’s restoration of daily function without further complications. This was the only case of a post-ECT fracture reported during 30 years of ECTs performed at our hospital, with COVID-19 regulations complicating fracture investigation.

Unmodified ECT refers to the administration of ECT without the prior administration of a muscle relaxant, as in this case. Succinylcholine is the most widely used muscle relaxant for modified ECT. Unmodified ECT was linked to an increased risk of fracture of vertebrae and long bones in 1954 [3]. The frequency of nonspinal musculoskeletal injury reported in the study was 3.5% (8/231 courses of ECT, were recorded as follows: fracture of the neck of humérus, (1); avulsion of lesser tuberosity of humérus (1); dislocation of the shoulder (2); dislocation of the mandible (2); tear of anterior tibial ligament (1); tear of shoulder capsule (1). However, the risks may be much smaller than this study showed, judging from more recent studies [9,19,20]. The use of unmodified ECT, in recent decades, remains debatable.

In one prospective study of 50 consecutive patients, who had received a course of six unmodified ECT treatments, routine radiological assessments identified only one (2%) patient to have experienced a minor, subclinical vertebral fracture [10]. In another prospective study of 56 consecutive patients, who received a mean of 2.9 unmodified ECTs as part of their ECT course, routine digital radiological assessments demonstrated that no patient experienced any spinal complication [21]. These findings contrast sharply with historical data that showed a 20–40% risk of dorsal spine vertebral body compression fractures with unmodified ECT [7,8]. Proposed reasons given for these differences include a slim body frame leading to less violent muscular contractions, youthfulness with an absence of risk of osteoporosis, physical restraints with less vigorous muscular movements, lower electrical charge, and the use of brief-pulse ECT versus sinusoidal wave ECT. [10].

Modification of ECT seizures using muscle relaxants requires the administration of general anesthesia, due to the adverse experience of full paralysis in patients while conscious. There is controversy regarding the dose of muscle relaxants given and a conservative attitude toward ECT practice in the operating room. A lower quantity reduced the apneic time and subsequent bag and mask ventilation during the COVID-19 pandemic [22,23]. However, for a variety of reasons, anesthesia cannot always be administered. The most common reasons are, the relative unavailability of qualified anesthesiologists, costs associated with modified ECT procedures, emergency and convenience, lack of equipment, contraindications in the use of succinylcholine, being ‘‘safer than a modified ECT’’, and it being reserved for young people [24,25]. 

More than six decades have passed since the introduction of succinylcholine-modified ECT [26]; nevertheless, it is apparent that unmodified ECT continues to be practiced. What might the reasons for this be? There are several explanations. The authors scrutinized some valuable articles regarding unmodified ECT without succinylcholine [20,27]. In Table 1, we show a variety of administrative and medical situations that should be taken into consideration when used in realistic circumstances in hospitals.

During the past seven decades, the use of unmodified ECT has continued to be reported from developing and developed countries, including African nations such as Nigeria [28,29], Uganda [30], and Malawi [31]; European countries such as the United Kingdom [32], France [33], Spain [34], and Russia [35]; and advancing or advanced Asian countries such as India [36], Turkey [37], Thailand [38], China [39], and Japan [25,40]. Unmodified ECT was still used in two-thirds of the hospitals in Japan between 2001 and 2003 [27].

A survey of the practice of ECT in Asia showed that an estimated 129,906 unmodified ECTs were administered to 55.7% of the patients in 141 (54.9%) institutions in 14 out of 23 countries between 2001 and 2003. These institutions included 62 university hospitals, 46 psychiatric hospitals, and 33 general hospitals. Whereas 93 (36.2%) of the institutions reported that they always administered unmodified treatments, 48 (18.7%) reported that they administered unmodified treatments between 1% and 98% of the time. The remaining 116 (45.1%) institutions reported that they never administered unmodified treatments [41]. If unmodified ECT is truly a dangerous procedure, there should be an epidemic of patients with orthopedic disabilities in the responding institutions, resulting in the termination of the practice of unmodified ECT. No such epidemic has been reported. These data suggest that even if unmodified ECT is associated with musculoskeletal risks, the risks are subclinical and not as large as historically recorded [41].

Shah N. et al. discussed Benzodiazepine-modified ECT, in relation to the administration of ECTs with an intravenous benzodiazepine (pretreatment with diazepam 10 mg, or lorazepam 4 mg, or midazolam 2 mg) administered in the past, in lieu of anesthesia and a muscle relaxant. No fractures were reported in this sample. Postictal confusion was rated at 10.7%. They suggested that Benzodiazepine-modified ECT is an acceptable alternative ECT for replacing completely unmodified treatment when such treatment is unavailable [21].

The authors have performed ECT with Thiamylal sodium alone (an ultra-short-acting barbiturate with less than 30 min duration of onset, such as thiopentone, and methohexitone), by intravenous injection. ECT was performed while the patient fell asleep without eyelash reflex. Seager (1959), in his study of 118 depressed patients, found similar therapeutic outcomes (the length of the treatment course, the length of stay in the hospital from the first day of ECT treatment, the period of recovery from the last treatment until discharge, and the total duration of stay in the hospital). This study covered patients receiving unmodified ECT, modified ECT with thiopentone and succinylcholine, or ECT modified with thiopentone alone [42].

We adopted a more flexible strategy for modified ECT. Some doctors invite anesthesiologists to perform modified ECT for elderly patients or patients with severe osteoporosis. In these cases, the psychiatrist, two nurses and one assistant escort the patient while moving their bed to the operating room in another building. Later, they accompany the patient back to the ward after the patient awakened. This entire process costs a lot of manpower and time. However, inpatients were not allowed to leave the ward during the COVID-19 pandemic. Almost all ECTs were administered smoothly by the psychiatric team in ECT rooms in the psychiatric ward.

## 4. Conclusions

The COVID-19 pandemic impacted multiple factors in the ECT treatment of, and subsequently investigation into, the post-ECT complications of this patient. Modified ECT procedures, with the use of muscle relaxants, is a dominant factor in maintaining safety to reduce the risk of musculoskeletal complications that may arise in many countries. The use of unmodified ECT continues to be reported by developing and developed countries for a variety of reasons. Psychiatrists should consider the risk of possible fractures when performing ECT. It is important to comply with COVID-19 regulations, but also to adopt a cautious and safe approach towards investigating and managing post-ECT complications, although ECT, without muscle relaxants, has a low risk of causing fractures.

## Figures and Tables

**Table 1:** Comparison of advantages of modified and unmodified electroconvulsive therapy (ECT).

	Modified ECT	Unmodified ECT
Administrative consideration		
Favored location for ECT treatment	In operational rooms	In psychiatric wards †
Facilities for anesthesiological practice	More	Less †
Participation of qualified anesthesiologist	Every ECT session	Teaching program †
Monopoly of anesthesiologists by surgical specialty	Possible	No †
Interest of anesthesiologists in minor procedure who are poorly remunerated	Need	No †
Infrastructure or equipment	More	Less †
Funding or cost (affordability of anesthesiological support)	More	Less †
Staffs’ ability to administer anesthesia	More †	Less
Medical consideration		
Urgent need for ECT (emergency and convenience)	Need arrangement	Easy †
Contraindications for use of anesthesia (e.g., because of cardiorespiratory disorders)	Safer by routine evaluation †	Consultation only for special cases needed
Contraindication for use of succinylcholine (e.g., in patients with burns, crush injuries, denervation syndromes, malignant hyperthermia or some neuromuscular disease)	Re-evaluation of muscle relaxants to prevent prolonged apnea [22,23]	No use, no concern †
Opinion of being “safer”	Less musculoskeletal complications †	From a survey of Japan report [25]
Intra-ECT blood pressure surge	Less possible †	More possible
Increased risk of loosened or broken teeth	Anecdotal report †	Maybe increased risk.
Increased risk of vertebrae compression fracture	Less possible †	20~40% in 1947 and 1950 [7,8], 2% in 2000 [9]
Increased risk of fractures of nonspinal bones or musculoskeletal injury	Less possible †	3.5% in the only report in 1954 [6]
Increased risk of uncomplicated backache or succinylcholine-induced myalgia	50% (1.5 to 89%) †	6.1 to 52%
Increased risk of restlessness and postictal confusion	Less †	More
Conceptually and visually unaesthetic image during a convulsion	No †	Yes

† Relatively favorable advantages.

## Data Availability

All data supporting our findings are contained within the manuscript.

## Abbreviations

ECT, Electroconvulsive therapy; SDM, Shared decision making; PHQ-9, Patient Health Questionnaire-9.
